# The Electrochemical Actuation Performances of Nanoporous Ternary AlCoCu Alloy with a Unique Nanosheet Structure

**DOI:** 10.3390/ma16216942

**Published:** 2023-10-29

**Authors:** Xiao Chen, Fuquan Tan, Jianfeng Wang, Kunpeng Zhao, Yaoguang Wang, Jie Zhang, Haixia Liu

**Affiliations:** 1 Shandong Provincial Key Laboratory of Molecular Engineering, School of Chemistry and Chemical Engineering, Qilu University of Technology (Shandong Academy of Sciences), Jinan 250353, China; 17864170661@163.com (X.C.); a1353007623@163.com (K.Z.); wangyaoguang9002@163.com (Y.W.); 2 Key Laboratory for Liquid-Solid Structural Evolution and Processing of Materials (Ministry of Education), School of Materials Science and Engineering, Shandong University, Jingshi Road 17923, Jinan 250061, China; z18779080029@163.com (F.T.); wangjianfeng@sdu.edu.cn (J.W.)

**Keywords:** electrochemical actuator, dealloying, nanoporous alloy, Al-Co-Cu alloy, strain amplitude, non-precious metal

## Abstract

Compared to traditional actuators (such as piezoelectric ceramics), metal actuators possess the advantages of a low energy consumption, large strain amplitude, and high strain energy density. However, most of the existing metal actuators with an excellent comprehensive performance are composed of precious metals, which are limited by high costs and have almost no possibility for large-scale production in the future. This study focuses on non-precious metal materials and exploits a one-step chemical dealloying method to prepare bulk nanoporous (NP) CoCuAl actuators (NP-CCA) from Al_70_Co_20_Cu_10_ alloy. The microstructure and actuation properties of the NP-CCA were analyzed in detail. The dense continuous nanoscale pores provide an excellent network connectivity for a large strain response, enabling the NP-CCA to achieve a strain amplitude of up to 1.19% (more than eight and two times that of NP-Pt and NP-Ag, respectively), comparable to precious metal actuators. In addition, the NP-CCA possesses a high strain energy density, which is prominent in many precious metal actuation materials (such as NP-Au, NP-Ag, and NP-Pt).

## 1. Introduction

The development of stable and low-cost actuation materials is a subject of concern in the field of actuation. At present, piezoelectric ceramics [1] and other actuation materials have been widely used, however, these materials have their own limitations. For example, piezoelectric ceramics and other non-metallic actuation materials [2] require a high potential to produce strain amplitude, which means that it is difficult to achieve a high sensitivity. With the discovery by the German scientist Jorg Weissmüller that pressed NP-Pt [3] produces considerable compensation stress and strain in a solid skeleton through changes in surface stress at a low potential, its function as an actuator was realized. NP-metals thus become a potential substitute for commercial actuators. Later, using a more convenient binary alloy dealloying method, a series of precious metal actuators, such as NP-Au [4,5,6], NP-Pd [7,8], and NP-Ag [9], were first designed. However, the raw materials of precious metal actuators are expensive and have limited reserves, making them unlikely to be put into mass production. In contrast, non-precious metals became substitutes for precious metals due to their relatively cheap prices and abundant reserves; currently, non-precious metal actuators such as NP-Cu [10,11] have been designed. However, non-precious metal actuators obtained through binary alloy dealloying are generally lower in terms of performance than noble metal actuators. In the face of this problem, a feasible solution is to increase the composition of binary alloys and design the precursor as a ternary alloy composed of non-precious metals, in order to improve its performance while maintaining a low cost, such as NP-AlNiCu [12] and nanoporous CuMnNi (NP-CMN) [13].

**Figure 1:**
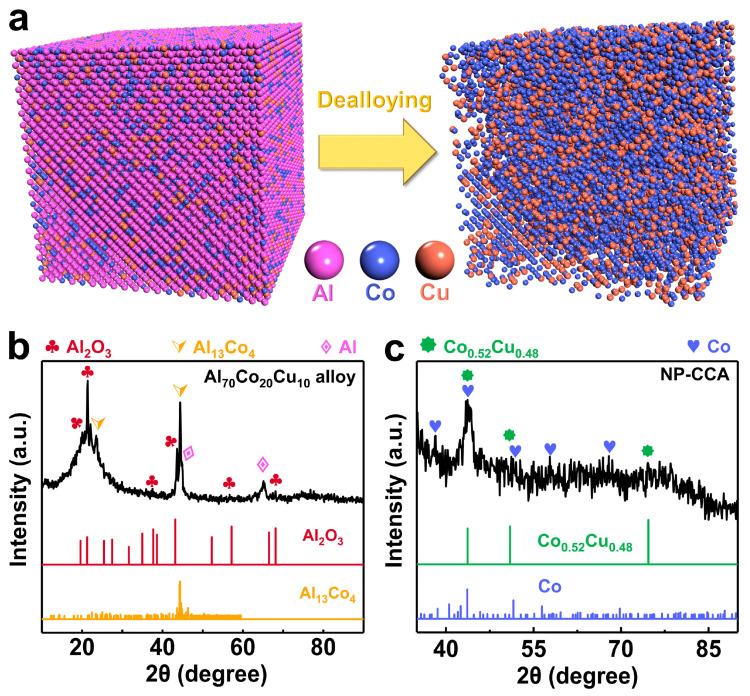
(**a**) Schematic diagram of the formation process of NP-CCA. XRD patterns of (**b**) Al_70_Co_20_Cu_10_ alloy ingot slices, and (**c**) NP-CCA.

The designed precursor alloy should be able to form a solid solution, because the atoms in the solid solution lattice are evenly distributed [14] and the nanoscale structure can be formed after dealloying [14,15]. Co and Cu are good metal actuation materials. Although the two non-precious metals exhibit different crystal structures, the atomic size is similar and the electronegativity difference is small, so they can form a solid solution [16,17]. Therefore, we designed a ternary AlCoCu alloy according to a certain mass ratio. Then, the precursor of AlCoCu was transformed into a nanoporous CoCuAl (NP-CCA) sample via a one-step chemical dealloying method (Figure 1a). During the dealloying process, the active element component Al is selectively corroded, while the remaining inactive element atoms Co and Cu are recombined [18], resulting in a large block of NP-CCA with a dense nanopore structure. The NP-CCA exhibits a large strain amplitude of up to 1.19%, exceeding NP-AlNiCu (0.9%), even better than many reported precious metal actuation materials, such as NP-Au [4] (0.24%) and NP-Ag [9] (0.50%). The Young’s modulus [19] and effective macroscopic Young’s modulus are at a higher level, which is obviously better than NP-AuPt [20]. Our results show that bulk NP-CCA with a dense nanopore structure possesses an excellent electrochemical actuating performance.

## 2. Experimental Section

### 2.1. Sample Preparation

Aluminum particles (99.99 wt.%), cobalt powder (99.99 wt.%), and copper sheets (99.99 wt.%) were placed in a graphite crucible at mass percentages of 51.02, 31.82, and 17.16%, and melted into an alloy solution with high-frequency induction heating (1550 ± 50 °C). The alloy solution quickly solidified into ingots, and was then cut into blocks. Then, the alloy was placed in a quartz tube with a small hole (the diameter was 0.8 mm) at the bottom, and was heated by an eddy current generated by an inductance coil, causing the alloy to quickly melt into a liquid state. Argon gas was conducted as the external positive air pressure, and the alloy liquid was blown into the copper mold (tubular, with an inner cavity size of 8 mm × 7 cm) through the small hole to make Al_70_Co_20_Cu_10_ alloy with a regular shape. The new Al_70_Co_20_Cu_10_ alloy was heated to 300 °C with a heating rate of 10 °C min^−1^ and this temperature was maintained for 12 h in the tube furnace to reduce the residual stress and stabilize the size. The alloy was cut into 1 × 1 × 1 mm^3^ cubes via a wire cutter. The Al_70_Co_20_Cu_10_ alloy cube was immersed in a 5 M NaOH solution, and dealloying was carried out at room temperature until no bubbles emerged. Then, the dealloying was conducted at 80 °C in the 5 M NaOH solution in order to further leach out the residual Al. Until no obvious bubbles appeared on the sample, the corrosion was stopped, and the solution was poured out to allow the sample to cool naturally. Then, the sample was soaked in deionized water for 3 h. After soaking, the sample was cleaned with deionized water several times, and dried in a vacuum drying oven at a constant temperature of 45 °C for 12 h.

### 2.2. Microstructure Characterization

The phase and element distribution of the polished precursor alloy surface was measured using back-scattered scanning electron microscopic (BS-SEM), combined with an energy-dispersive X-ray spectrometer (EDX). The microstructure and morphology of NP-CCA were characterized using a field emission scanning electron microscope (SEM, JSM-7610F). The phase identification of the precursor alloy and NP-CCA sample was performed using an X-ray diffractometer with Cu Kα radiation (XRD, XD-3, Beijing Purkinje General Instrument Co., Ltd., Beijing, China). Using ImageJ software (Version 1.38), 2100 nanoholes in the SEM images were statistically processed.

### 2.3. Actuation Measurements

The electrochemical actuation experiments were performed in a three-electrode system at room temperature in the potential range of −0.8~+0.5 V, using 1 M NaOH as the electrolyte. The schematic diagram of the actuation device is shown in Figure S1. The NP-CCA placed on the Au sheet was connected to the potentiostat by the Au wire. The quartz pushrod contacted the upper surface of the sample and the tip of the fixed displacement sensor (Solartron, DP/1/S) simultaneously. The sample would expand or contract accordingly with the potential change provided by the potentiostat (CHI 760E). The longitudinal displacement of the sample was converted into a digital signal by the sensor, which was transmitted to the computer and recorded. The NP-CCA sample on a Au sheet was used as the working electrode (WE), while a saturated calomel electrode (SCE) and carbon paper were used as the reference electrode (RE) and counter electrode (CE), respectively.

## 3. Results and Discussion

### 3.1. Microstructures of Al-Co-Cu Precursor Alloy and NP-CCA

The phase compositions of the precursor alloy Al_70_Co_20_Cu_10_ and NP-CCA were analyzed using XRD. Figure 1b shows the XRD pattern of the precursor alloy. The characteristic peaks are well correlated with α-Al (PDF # 65-2869), Al_2_O_3_ (PDF # 50-1496), and Al_13_Co_4_ (PDF # 65-1165). After immersion in a 5 M NaOH solution for dealloying, the possible reaction equation is [21]:

(1) $$2\mathrm{Al}+2\mathrm{NaOH}+2H_{2}O=2{\mathrm{NaAlO}}_{2\;}+3H_{2}$$

**Figure 2:**
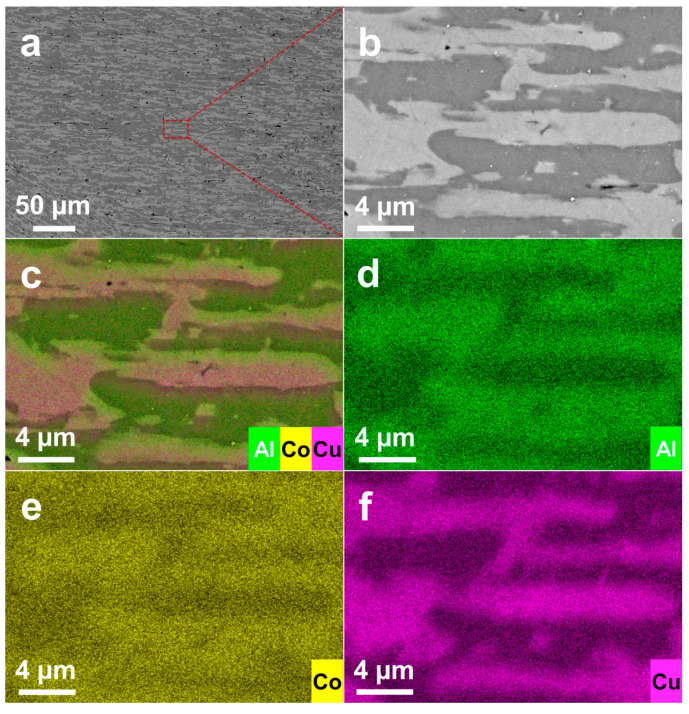
(**a**,**b**) BS-SEM images of alloy ingots. EDX mapping results of (**c**) Al + Co + Cu, (**d**) Al, (**e**) Co, and (**f**) Cu associated with (**b**).

NaOH solution was used to selectively corrode the Al phase in the alloy and the Al element in the Al_13_Co_4_ phase, while the remaining inactive Co and Cu atoms recombined and formed the Co (PDF # 65-9722) and Co_0.52_Cu_0.48_ (PDF # 50-1452). The corresponding peaks can be found in Figure 1c. The BS-SEM images of the alloy precursor after surface polishing are shown in Figure 2a,b. The EDX results of the Al_70_Co_20_Cu_10_ precursor alloy (Figure 2c) illustrate the coexistence of Al, Co, and Cu elements, indicating the formation of an AlCoCu alloy. A portion of the Co atoms in the Al_13_Co_4_ lattice were replaced by Cu atoms. Therefore, the phase type of the Al_13_Co_4_ was designated as Al_13_(Co,Cu)_4_. After removing Al_2_O_3_ from the surface by polishing, two main structures/phases existed in the alloy, which were a dark primary Al phase and gray Al_13_(Co,Cu)_4_ dendrites. The EDX results (Figure 2d–f) also confirm the presence of Al and Al_13_(Co,Cu)_4_ in dark and gray regions, respectively. Al_13_(Co,Cu)_4_ is mostly surrounded by Al, indicating that Al_13_(Co,Cu)_4_ is formed by a peritectic reaction [22], and the α-Al phase permeates with Al_13_(Co,Cu)_4_. Figure S2a shows the proportion of elements on the alloy surface, and the measured element ratio is Al_68.9_Co_19.6_Cu_11.5_, which is close to the theoretical composition of the Al_70_Co_20_Cu_10_ alloy. Figure S2b shows the proportion of elements on the surface of NP-CCA. The results show that Al elements are not completely removed from the Al_70_Co_20_Cu_10_ precursor alloy, and the residual Al content accounts for about 6.0% of NP-CCA. The BS-SEM images (Figure S3a–d) of NP-CCA show the coexistence of Al, Co, and Cu elements.

**Figure 3:**
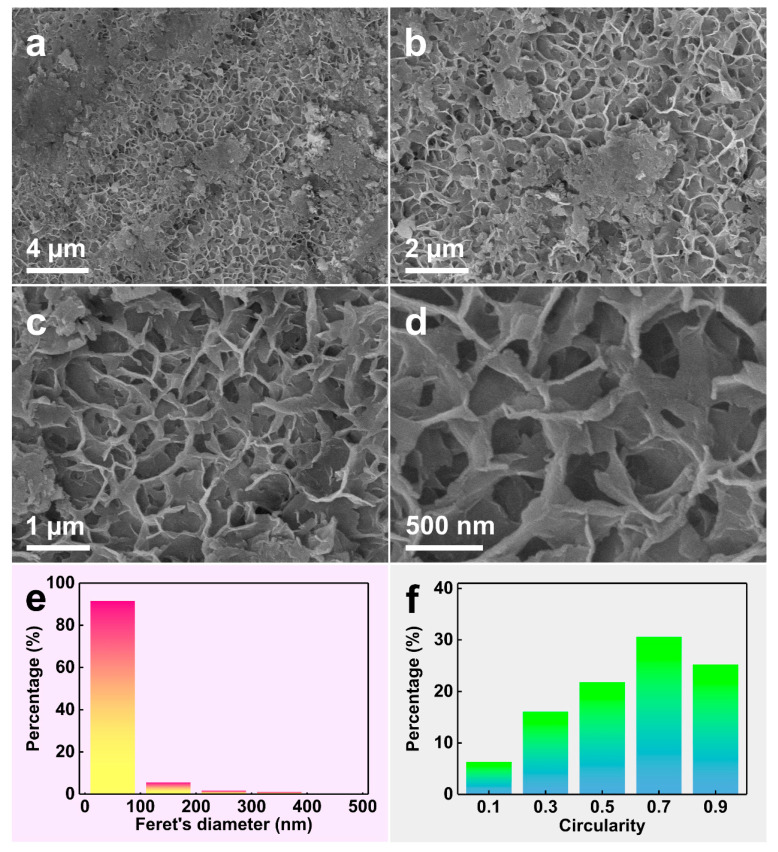
(**a**–**d**) SEM images of the NP-CCA surface at different magnifications. (**e**) Histograms of the Feret’s diameter and (**f**) circularity distribution of the nanopores on NP-CCA surface.

**Figure 4:**
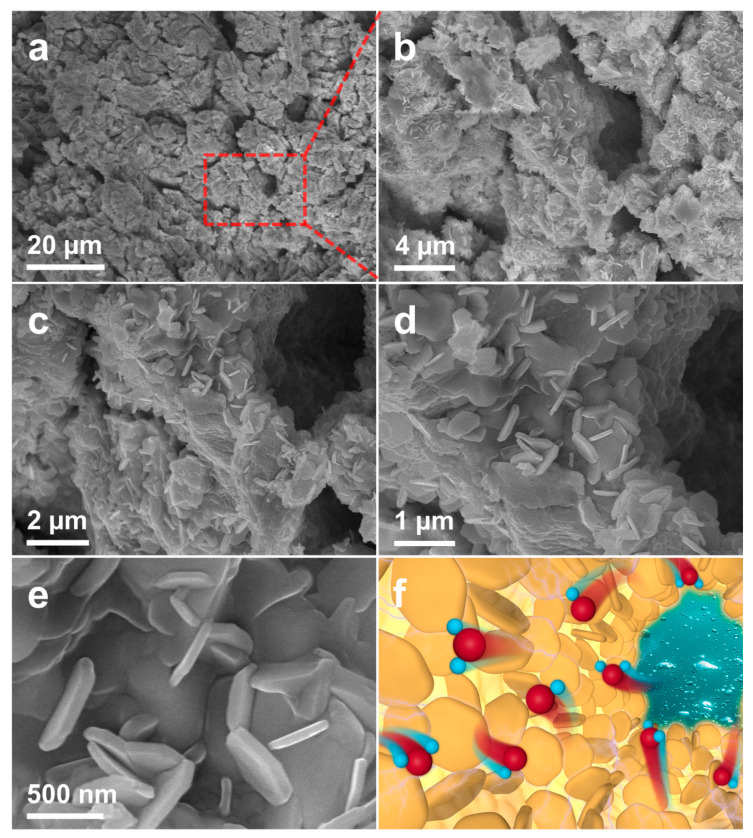
(**a**–**e**) SEM images of the interior of NP-CCA at different magnifications. (**f**) Schematic diagram of the electrochemical actuation process.

Figure 3a–d display the SEM images of the dealloyed samples at different magnifications, from which the nanosheet structure and surrounding nanopore structure can be observed clearly. In order to study the regularity of these nanopores, the concepts of Feret’s diameter [23] (the distance between the two boundary parallel lines on the nanopore projection profile) and circularity [24] (the proximity of nanopore projection to standard circles) were introduced, and 2100 SEM images of the nanopore surface were analyzed using the ImageJ software (Version 1.38). The calculated average Feret’s diameter was 82 nm. Figure 3e also shows that the Feret’s diameter of the sample was concentrated in the range of 0~100 nm, indicating that the size of the nanopore was uniform. The definition domain of circularity was between 0 and 1. The closer the projection shape of the nanopore is to a circular shape, the closer its circularity value is to 1. Conversely, the closer its circularity value is to 0. Figure 3f shows the distribution of the circularity of the sample. The average circularity of the sample was 0.79 after calculation, indicating that the shapes of the nanopores on the surface of the sample were relatively regular. The microstructures inside the sample can be obtained from the cross-view SEM images by breaking the sample. Figure 4a–e shows the cross-view SEM images of the NP-CCA sample at different magnifications. It can be observed that the inner wall of the sample is stacked with a large number of hexagonal nanosheets [25], forming a unique hexagonal nanosheets/nanopore structure.

### 3.2. Electrochemical Actuation Properties of the NP-CCA Sample

#### 3.2.1. Electrochemical Actuation Stability Test

The actuation behavior discovered in NP-Pt in 2003 has given NP-metals the potential to become metallic artificial muscle [26]. The electrochemical actuation mechanism of NP-metals originates from their charge-induced strain property. In general, the surface stress of these materials can be adjusted by changing the free electron density at the interface of NP-metals with a high-surface-to-volume ratio. Due to the high-surface-to-volume ratio of NP-metal, a change in its surface stress, in turn, causes a detectable macroscopic reversible strain in its body, manifested as expansion or contraction behavior [27]. The in situ dilatometry method [28] was used to determine the electrochemical actuation behaviors of NP-CCA. Figure 4f shows the schematic diagram of the electrochemical actuation process at the microscopic level, in which red and blue pellets represent oxygen and hydrogen atoms, respectively.

**Figure 5:**
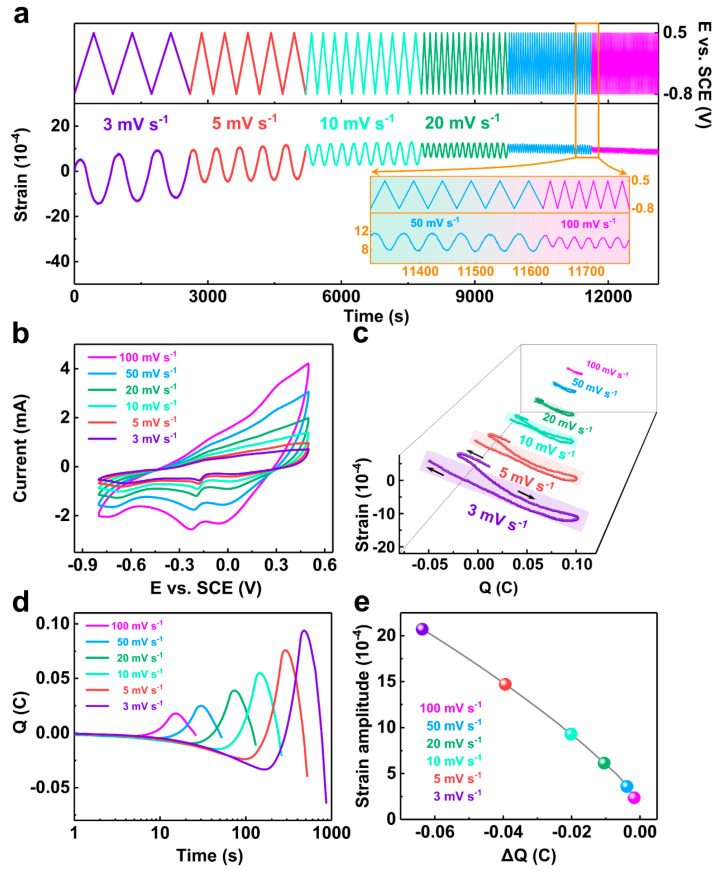
(**a**) Continuous actuation response and local magnification of NP-CCA under CV induction in 1 M NaOH solution, (**b**) CV curves, (**c**) strain–charge curves, (**d**) transfer charge versus time, and (**e**) the relationship between strain amplitude and the total amount of transferred charges at different scan rates.

The NP-CCA is first placed in the electrolyte for a period of time, waiting for the strain curve to flatten out (Figure S4a). Cyclic voltammetry (CV) [29] is used to provide electrochemical stimulation for the actuation measurement of the NP-CCA sample. Figure S4b shows the CV pretreatment results of NP-CCA in a 1 M NaOH solution. The strain curve of the sample eventually becomes reversible with an increase in CV cycles. At the same time, the CV curve of the sample also maintains a good repeatability, which can be observed from Figure S5a and its three-dimensional diagram of Figure S5b. The linear strain [30] is defined as *ε =* Δ*L/L_0_*, where *L_0_* is the original length of the sample and *ΔL* is the change in the length of the sample [31,32]. Figure 5a shows the reversible actuation response of NP-CCA samples in the 1 M NaOH solution at the scan rate of 3~100 mV s^−1^. It can be seen that all the strain responses are stable and reversible, as shown by the strain-E curves in Figure S6a. Figure S6b shows that the strain amplitude of NP-CCA gradually decreases from 0.2 to 0.02% with an increase in scanning rate. This suggests that the strain amplitude is negatively correlated with the scan rate, which can be attributed to the complex nanopore structure limiting the diffusion process of related substances such as OH^−^ at high scan rates [33]. Figure 5b shows the CV curves with a scanning rate of 3~100 mV s^−1^. The anode [34] and cathode peaks that satisfy the electron neutralization during the redox reaction move to the directions of positive and negative potential, respectively, with an increase in the scan rate, owing to the limitation of the diffusion rate of the OH^−^ form.

#### 3.2.2. Capacitive Capacity of Materials

It is well known that the electrochemical actuation of porous metal materials results from large amounts of stress-induced compensated stress/strain in their solid skeleton. Figure S6c shows the current–time curve, and the charge *Q* can be obtained by integrating the current–time curve. Here, we introduce the stress–charge coefficient (*ζ*) to describe the linear relationship between induced strain and transferred charge [13]. Figure 5c shows the relationship between the reversible strain and charge of an oxide-covered surface for the NP-CCA sample. The relationships between the stress–charge coefficient and macroscopic strain (Δ*L/L_0_*), transfer charge (Δ*Q*), and sample mass m are shown in Equation (2):

(2) $$\zeta =-\frac{9\mathrm{Km}}{2\rho }\frac{\Delta L/L_{0}}{\Delta Q}$$

where *K* and *ρ* represent the bulk modulus and mass density of the NP-CCA sample, respectively (171.6 GPa and 2.76 g cm^−3^ in Table S1). The value of (Δ*L*/*L*_0_)/Δ*Q* can be obtained by linear fitting the strain-*Q* curve [12], which can be approximated as the slope of the curve, represented as the slope of the transparent rectangle in Figure 5c. It is clear that the reversible strain has a linear negative correlation with the transferred charge, that is, an increase in the transferred charge corresponds to the contraction of the sample. The strain-charge coefficient of the detected NP-CCA sample ranges from +8.4 to +10.7 V, and the values are consistent with those reported in other metals with oxide layers (also exhibit positive values) [28,35,36].

Figure 5d shows the relationship between *Q* and time. The *Q* values [37] increase from +0.020 to +0.157 C when the scan rate decreases from 100 to 3 mV s^−1^, which means that a lower sweep speed can transfer more charge. The maximum change in stress value presents an approximate linear relationship with the total transferred charge value (Figure 5e). More transferred charge means a larger surface stress, indicating that a large stress–strain value also depends on the capacitive capacity of the material [38].

The CV curve in Figure S6d shows the characteristic peak of OH^−^ adsorption/desorption (OA/OD) during the CV test of NP-CCA. The adsorption and desorption peaks are located at −0.33 and +0.15 V, respectively, at 2 mV s^−1^. Possible redox reactions are as follows [39,40]:

(3) $$\mathrm{CoO}+{OH}^{-}=\;\mathrm{CoOOH}+e^{-}$$

(4) $$\mathrm{CuO}+{OH}^{-}=\;\mathrm{CuOOH}+e^{-}$$

At higher scanning rates, the strain amplitude gets smaller due to the reduction in stored charge at the faster charging rate. At the first CV cycles, Co oxides grow spontaneously on the surface of NP-CCA, which helps to stabilize the pore structure and prevent it from coarsening, resulting in a stable and reversible actuation.

#### 3.2.3. Maximum Strain Amplitude

**Figure 6:**
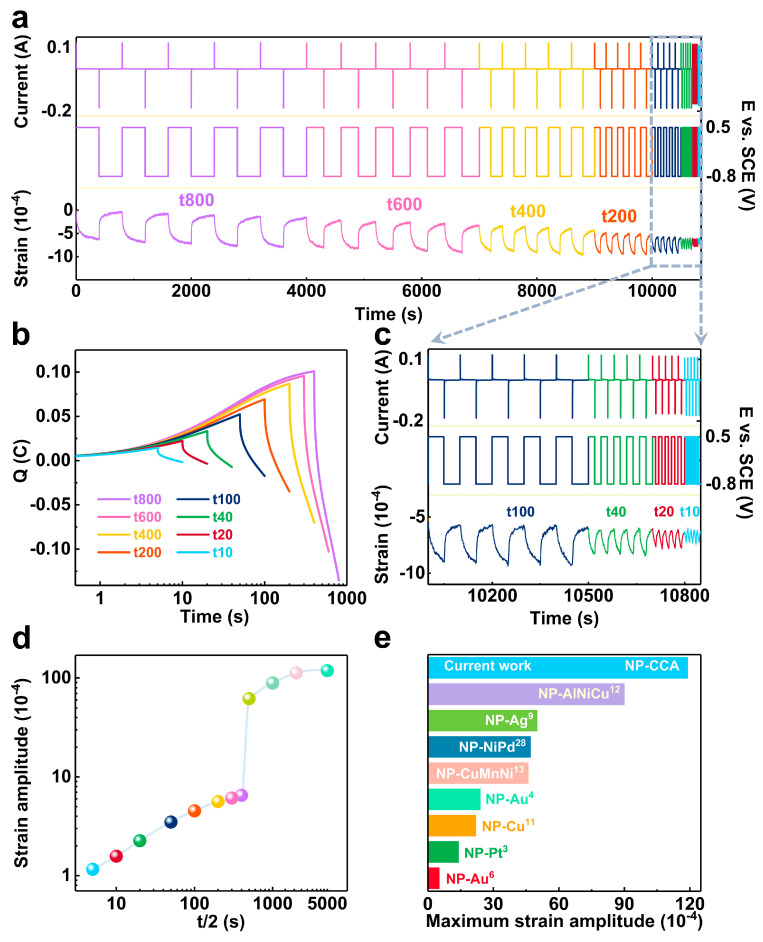
(**a**) CA-induced continuous actuation response of NP-CCA in 1 M NaOH solution at different periods, with potential switching between +0.5 and −0.8 V vs. SCE repeatedly. (**b**) The relationship between transfer charge and time under different periods. (**c**) An enlarged view of the dashed area in Figure 6a. (**d**) The relationship between strain amplitude and half-period of charge/discharge. (**e**) Bar chart comparison of maximum strain amplitudes for different NP-metals/alloys.

To further explore the maximum strain amplitude of NP-CCA, chronoamperometry (CA) [41] was operated on the sample, which can ensure the full and rapid adsorption of OH^−^ at +0.5 V vs. SCE and the desorption of OH^−^ at −0.8 V vs. SCE, as shown in Figure S7a. Figure 6a shows the reversible strain response of the NP-CCA sample to CA stimulation in a series of periods, and the strain amplitude increases with an extension of the charging time. By integrating the current curve of a single period in Figure 6a, the relationship between transferred charge and time can be obtained under different periods (Figure 6b). The *Q* value increases from +0.016 to +0.236 C with the continuous extension of the period. Figure 6c is an enlarged view of the dashed region in Figure 6a, from which the stable and reversible strain response of the sample can be observed. The amount of OH^−^ that can be adsorbed is limited under the stimulation of +0.5 V, so the strain amplitude gradually reaches the upper limit after the cycle time reaches a certain value. The sample reaches the maximum strain amplitude of 1.19% (Figure 6d) when the period reaches 10,000 s, which is five times that of NP-Cu (0.22%) [11]. In addition, the strain rate is a key issue for the metallic actuator [12], which is mainly restricted by the mass transfer within the nanopore of the bulk metallic actuator. The strain rate can be obtained by dividing the strain amplitude by the corresponding half-period (T/2). Figure S7b shows that the strain rate gradually decreases with an increase in the charging time, and a turning point occurs at T/2 = 500 s, owing to the sharp increase in the strain amplitude (according to Figure 6d). The maximum strain rate of NP-CCA sample is 2.3 × 10^−5^ s^−1^. The high strain rate originates from the unique microscale channel formed after the removal of α-Al, favoring the transmission of electrolyte inside and outside the sample during the electrochemical actuation process.

#### 3.2.4. Comparison of Actuation Performance with Other NP-Metals

Figure 6e lists the maximum strain amplitudes of various nanometal/alloy actuators, and our NP-CCA samples composed of non-precious metals exhibited large strain amplitudes that even exceed those of some precious metal actuators. The strain energy density [42] of NP-metals is an important parameter for actuation material and can evaluate the strength, toughness, and service life of the material. If the actuator appear as a linear elastic solid [43], the volumetric specific strain energy density is given by Equation (5):

(5) wV= 12Yeffεmax2

where *Y*_eff_ is the effective macroscopic Young’s modulus and *ε*_max_ is the maximum reversible strain. Furthermore, the mass specific strain energy density [12] can be obtained from Equation (6):

(6) $$w_{M}=w_{V}/\rho$$

where *ρ* is the mass density of the actuation material. At present, some key parameters of bulk nanomaterials, such as the elastic constant [44] or yield strength [45], are still blank fields. Therefore, the NP-CCA is treated as an open-cell foam material [46], and the modulus *Y*_eff_ can be obtained from Equation (7):

(7) $$Y_{\mathrm{eff}}=Y_{s}{\left(\rho_{\mathrm{NP}}/\rho_{s}\right)}^{2}$$

where *Y*_s_ is the Young’s modulus of solid metal and *ρ*_np_ and *ρ*_s_ are the densities of NP-metal/alloy and solid metal [10], respectively. *ρ*_NP_/*ρ*_s_ is the solid phase fraction (*φ*) of NP-metal in this paper, and it can be obtained from the porosity (*φ*_p_) (67.1%) [8]. For simplicity, we consider the Young’s modulus (bulk modulus) of an NP alloy as the sum of the product of the Young’s modulus (bulk modulus) of each metal and its relative proportion in the alloy [12]. Table S2 lists the Young’s moduli of *Y*_s_ (alloy) and *Y*_s_ (metal) of the alloy. The values of *Y*_s_, *φ*, *Y*_eff_, *w*_V_, and *w*_M_ of the sample are 249.1 Gpa, 32.9 × 10^−2^, 14.0 Gpa, 991.3 kJ m^−3^, and 359.17 kJ m^−3^, respectively. Figure S8 compares the above parameters between NP-CCA and NP-AuPt, and the specific values of these parameters can be found in Table S3. The values of the maximum strain amplitude and volume specific strain energy density of our NP-CCA are close to those of NP-AuPt, but the Young’s modulus and effective macroscopic Young’s modulus are significantly higher than those of NP-AuPt, and the material cost is greatly reduced.

## 4. Conclusions

An AlCoCu ternary alloy was designed according to a certain mass ratio. After dealloying, NP-CCA with a unique micromorphology was obtained, which exhibited an excellent stability during electrochemical actuation performances in alkaline electrolyte. The strain amplitude could reach 1.19%, which is more than eight and two times that of NP-Pt and NP-Ag, respectively. The maximum strain rate was 2.3 × 10^−5^ s^−1^, indicating that it was very sensitive to electrochemical stimulation. In summary, NP-CCA displayed a significant actuation performance, high alkali resistance, and low material cost, and is a potential actuation material. This study provides a new idea for material design in the field of sensing, especially in the field of electrochemical actuation.

## Data Availability

In this paper, all the data, models, and code used during the study appear in the submitted article.

## Supplementary Materials

The following supporting information can be downloaded at: https://www.mdpi.com/article/10.3390/ma16216942/s1, Calculation Formulas (1)–(4) provides relative density, mass density, porosity and *K* value of the as-dealloyed samples; Figure S1: Schematic illustration of the actuation device with three electrodes; Figure S2: Typical EDX spectra of (a) alloy ingot sections, and (b) NP-CCA surfaces. The corresponding composition is listed in inset of (a) and (b); Figure S3: EDX-mapping images of (a) Al+Co+Cu, (b) Al, (c) Co, and (d) Cu in the NP-CCA sample; Figure S4: (a) The strain curve of the NP-CCA sample under static state. (b) The strain curve of the NP-CCA sample was pre tested at a scan rate of 100 mV s^−1^ before formal testing; Figure S5: (a) 2D images and (b) corresponding 3D waterfall plots obtained by equidistant sampling of the CV curves of NP-CCA sample at a scan rate of 100 mV s^−1^; Figure S6: (a) Strain and E relationship diagram. (b) Strain amplitude versus the scan rate. (c) The current-time relationship diagram of NP-CCA at different scan rates, and (d) the actuation response curves of the NP-CCA sample for two cycles at 2 mV s^−1^; Figure S7: (a) The actuation response curve of the NP-CCA sample at a period of 1000 s. (b) The relationship between strain rate and half-period of charge/discharge; Figure S8: Radar chart of performance comparison between NP-CCA and NP-AuPt; Table S1: Stress charge coefficient and other parameters of NP-CCA at different scan rates; Table S2: The Young’s modulus of NP-CCA was obtained based on the Young’s modulus of Al, Co, and Cu metals. The *Y*s value for each dealloyed sample can be estimated by such an equation: *Y*(*Di*) = *∑* *X*(*M*)**Y*(*M*), (*M* is Al, Co and Cu); Table S3: Various parameters of NP-CCA and NP-AuPt. Note that the strain energy density is in linear values. References [12,47] are cited in the Supplementary Materials.
