# Supplementary material for: The Electrochemical Actuation Performances of Nanoporous Ternary AlCoCu Alloy with a Unique Nanosheet Structure
Source: Materials (Basel). 2023 Oct 29;16(21):6942. doi: 10.3390/ma16216942 (PMC10648953; doi:10.3390/ma16216942)
Supplement: Supplementary file 1 [file materials-16-06942-s001.zip › materials-2681788-supplementary.pdf]

Supporting Information

The Electrochemical Actuation Performances of Nanoporous Ternary AlCoCu Alloy with a Unique Nanosheet Structure

Xiao Chen [1], Fuquan Tan [2], Jianfeng Wang [2], Kunpeng Zhao [1], Yaoguang Wang [1], Jie Zhang [1,*] and Haixia Liu [1,*]

[1] Shandong Provincial Key Laboratory of Molecular Engineering, School of Chemistry and Chemical Engineering, Qilu University of Technology (Shandong Academy of Sciences), Jinan 250353, China

[2] Key Laboratory for Liquid-Solid Structural Evolution and Processing of Materials (Ministry of Education), School of Materials Science and Engineering, Shandong University, Jingshi Road 17923, Jinan 250061, China

* Correspondence: zh_jie@qlu.edu.cn (J.Z.); liuhaixia929@163.com (H.L.)

This part includes:

1. Calculation formulas (Formulas 1-4)
2. Figures (Figure S1-S8)
3. Tables (Table S1-S3)
4. References

1. Calculation formulas (Formulas 1-4)

1.1. Calculation of the relative density of NP-CCA samples

The relative density of the as-dealloyed samples can be calculated as follows:

$$\varphi = \frac{\rho_{NP}}{\rho_s} \times 100\% \qquad \text{(S1)}$$

where the mass density ρ_{NP} of the NP-CCA sample can be determined from the sample mass divided by its volume, and the mass density ρ_s of the solid alloy with the same composition as the as-dealloyed sample can be calculated by the equation:

$$\rho_s = \frac{1}{\sum \frac{W(\mathrm{M})}{\rho(\mathrm{M})}} \qquad \text{(S2)}$$

where the W is the mass percentage, which is the density of pure metal of Al, Co and Cu. Therefore, the relative densities for NP-CCA were calculated to be 32.9 %.

The porosity (φ_P) of the NP-CCA can be obtained by the equation:

$$\varphi_p = 1 - \varphi \qquad \text{(S3)}$$

1.2. Calculation of the *K* value of NP-CCA

At present, there are few literatures on determining *K* value for porous alloys, and many researchers focus on single-element porous metals. Therefore, the *K* value was estimated to be the sum of the product of the metal's bulk modulus [1,2] and their relative percentage ratio for the NP-CCA alloys, that is,

$$K=\sum X(\mathrm{M})*K(\mathrm{M}) \tag{S4}$$

where the *X* is the atomic percentage, M is Al, Co and Cu. The *K* values of the NP-CCA were determined to be 171.6 GPa.

2. Figures (Figure S1-Figure S8)

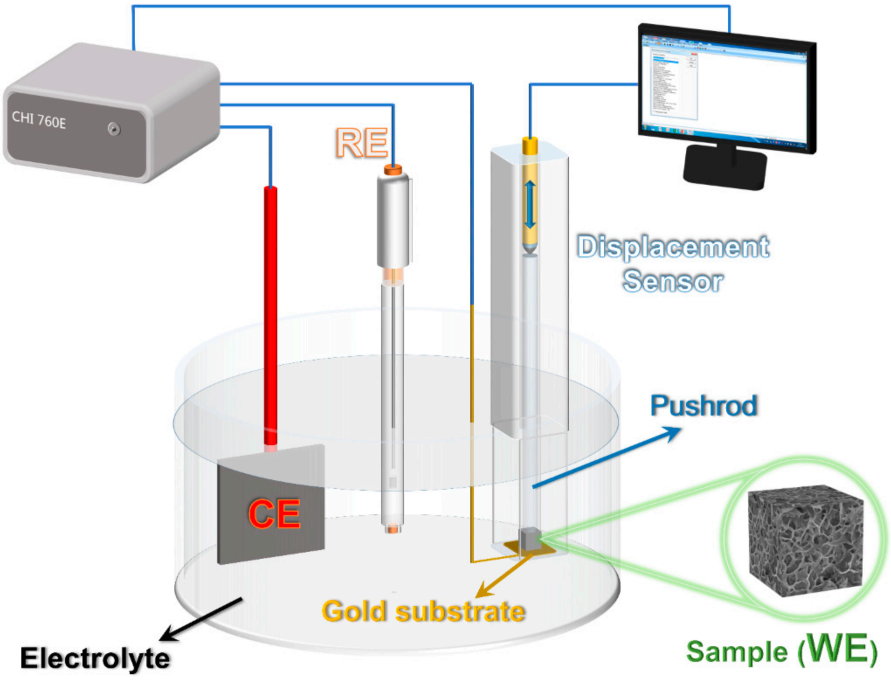

Figure S1. Schematic illustration of the actuation device with three electrodes.

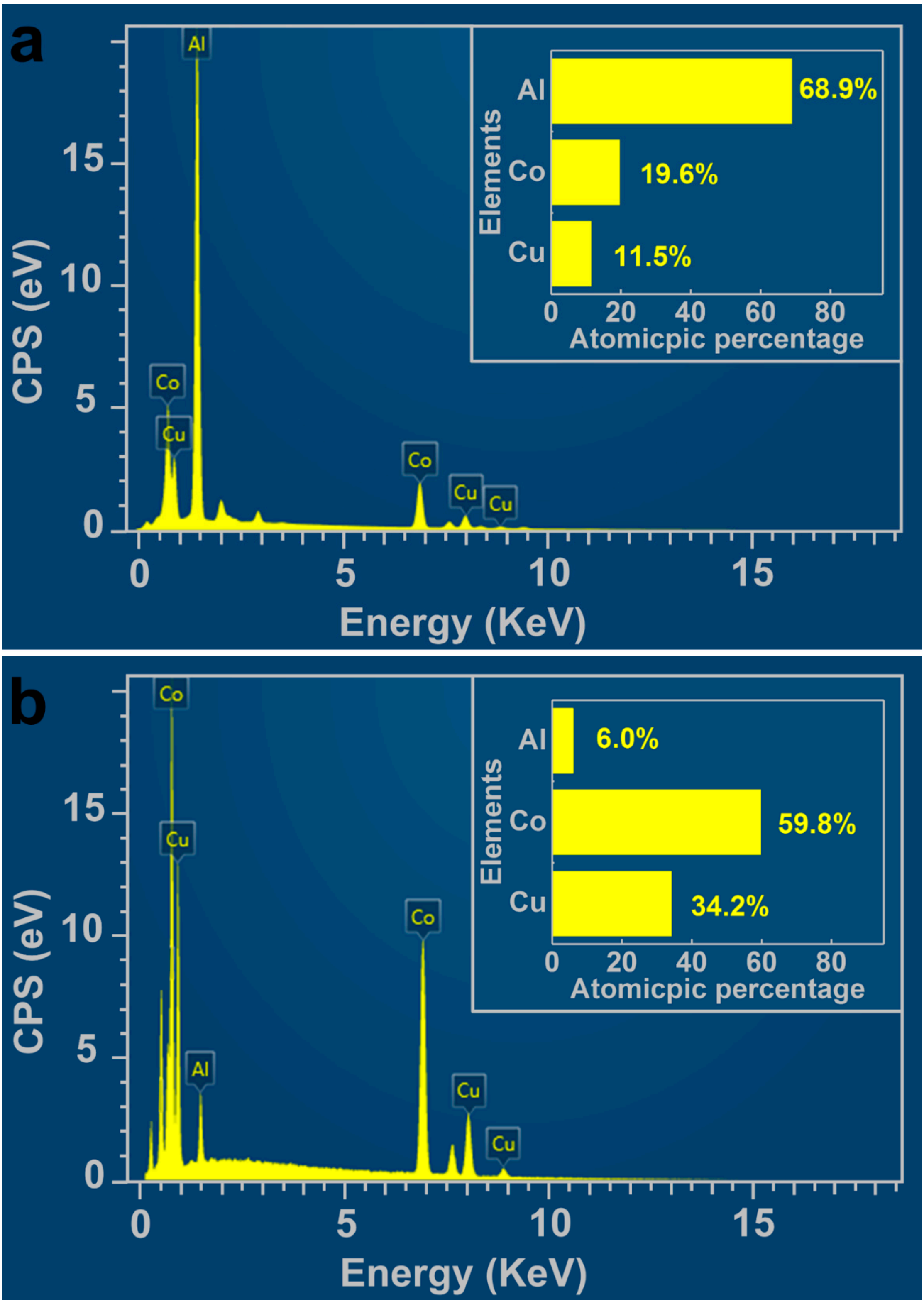

Figure S2. Typical EDX spectra of (a) alloy ingot sections, and (b) NP-CCA surfaces. The corresponding composition is listed in inset of (a) and (b).

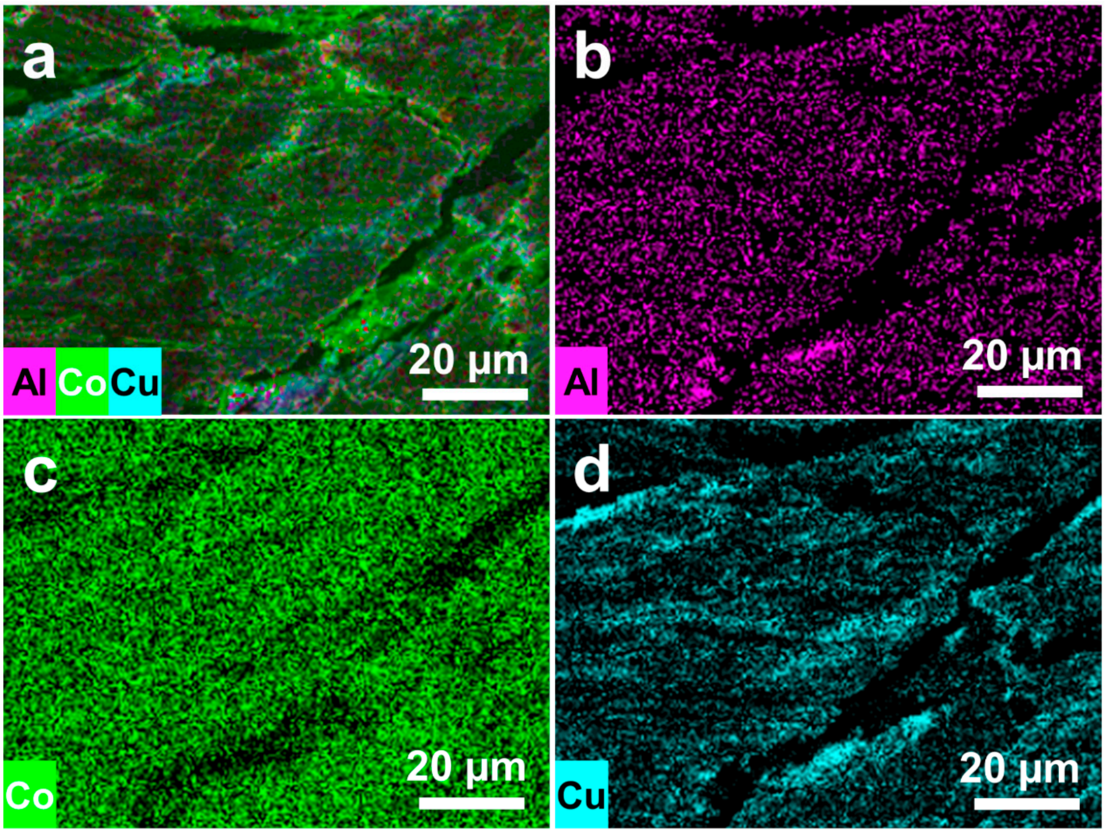

Figure S3. EDX-mapping images of (a) Al+Co+Cu, (b) Al, (c) Co, and (d) Cu in the NP-CCA sample.

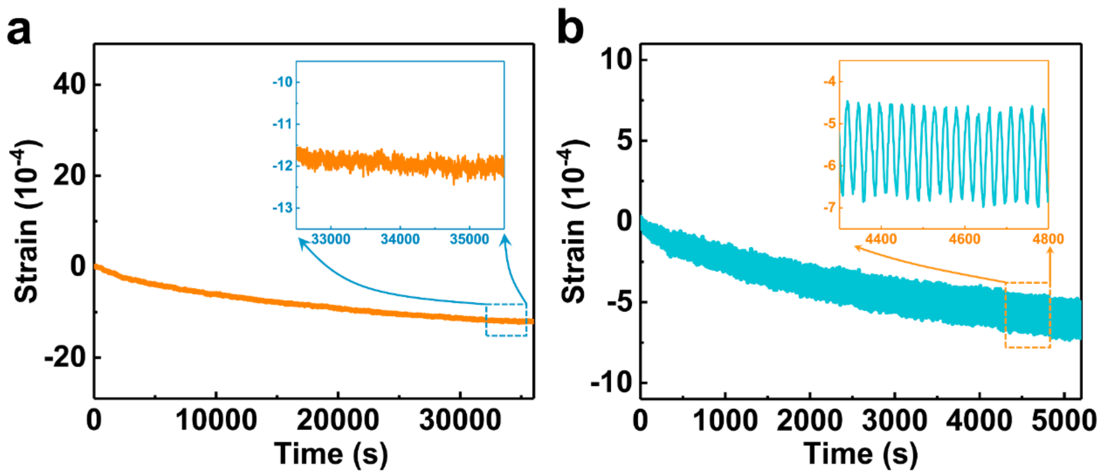

Figure S4. (a) The strain curve of the NP-CCA sample under static state. (b) The strain curve of the NP-CCA sample was pre tested at a scan rate of 100 mV s^{-1} before formal testing.

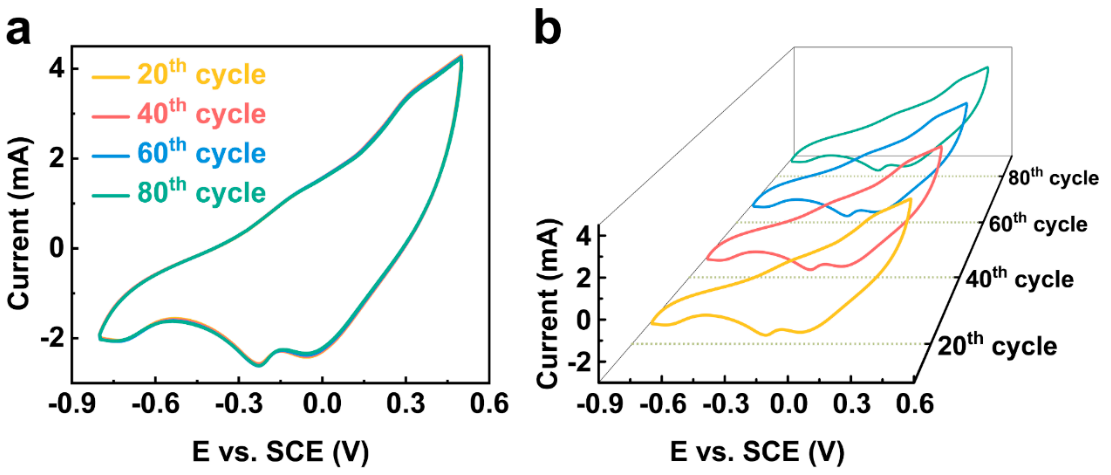

Figure S5. (a) 2D images and (b) corresponding 3D waterfall plots obtained by equidistant sampling of the CV curves of NP-CCA sample at a scan rate of 100 mV s^{-1}.

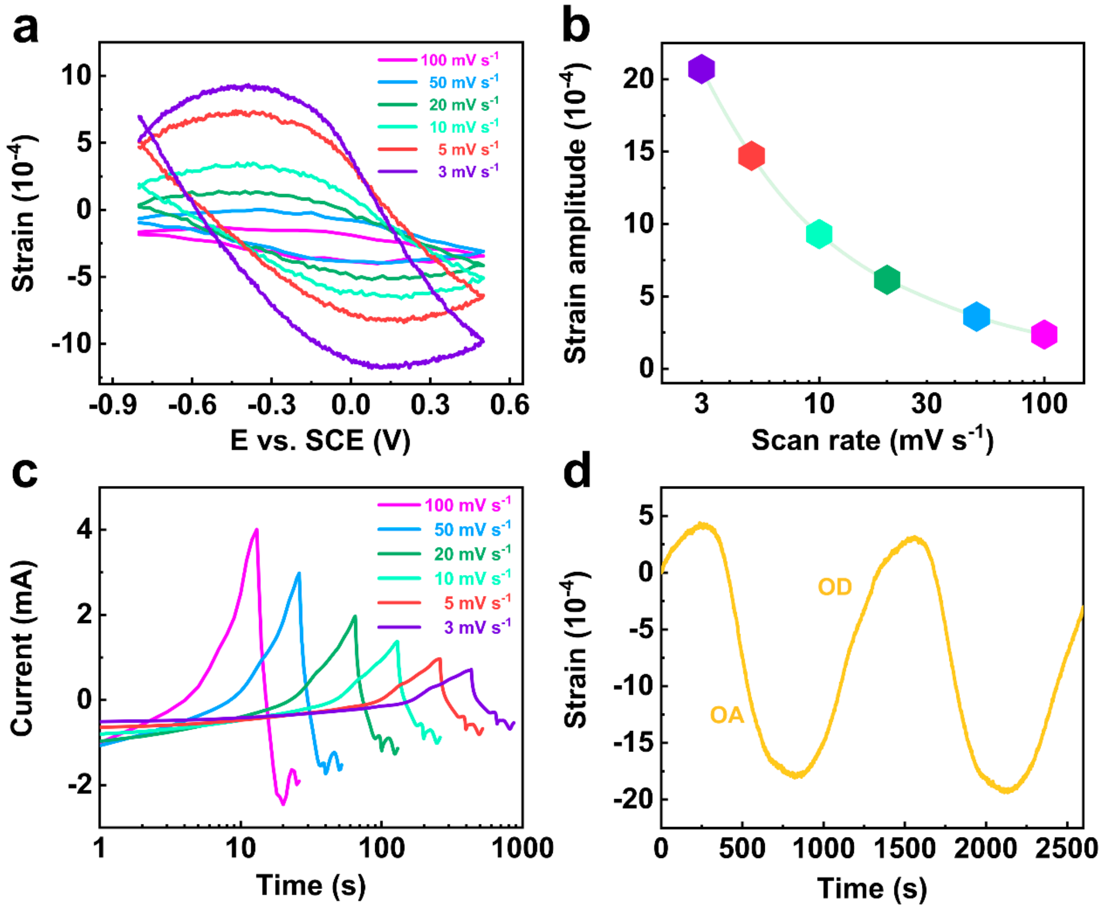

Figure S6. (a) Strain and E relationship diagram. (b) Strain amplitude versus the scan rate. (c) The current-time relationship diagram of NP-CCA at different scan rates, and (d) the actuation response curves of the NP-CCA sample for two cycles at 2 mV s^{-1}.

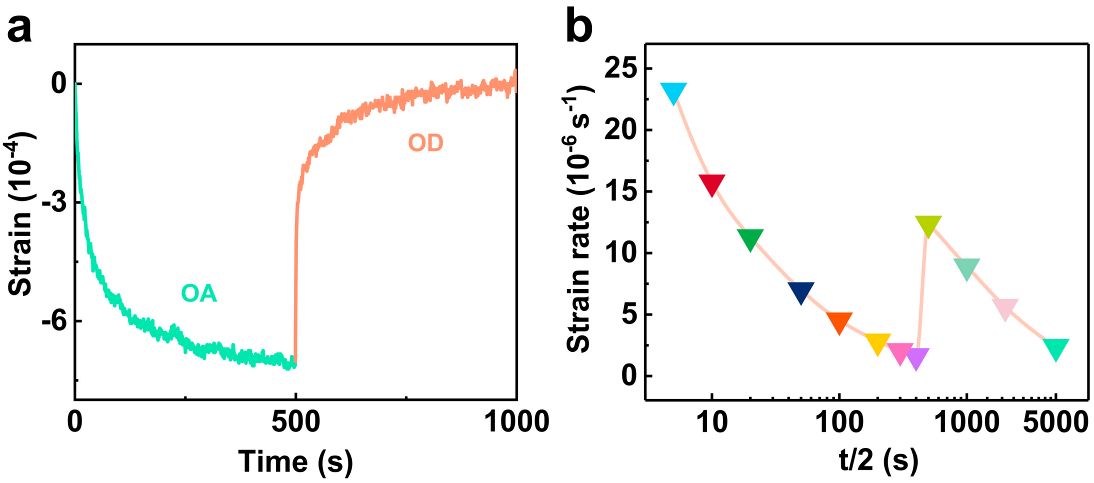

Figure S7. (a) The actuation response curve of the NP-CCA sample at a period of 1000 s. (b) The relationship between strain rate and half-period of charge/discharge.

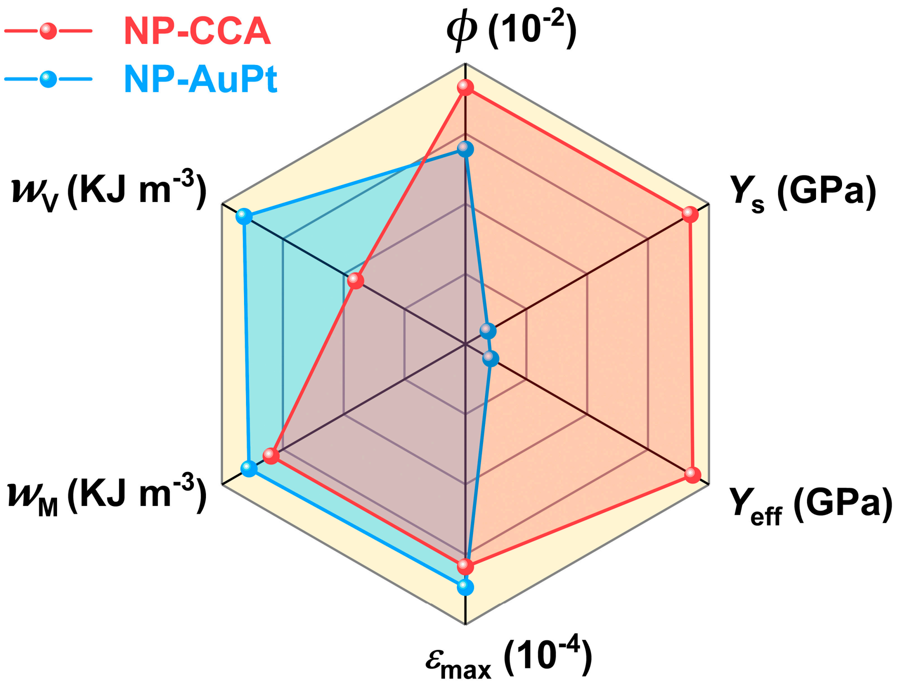

Figure S8. Radar chart of performance comparison between NP-CCA and NP-AuPt.

3. Tables (Table S1-Table S3)

Table S1. Stress charge coefficient and other parameters of NP-CCA at different scan rates.

**Scan rate (mV s^{-1})**	***X*(Al) (at.%)**	***X*(Co) (at.%)**	***X*(Cu) (at.%)**	**K(Al) (GPa)**	**K(Co) (GPa)**	**K(Cu) (GPa)**	**Estimated K (GPa)**	**ρ (g/cm^3)**	**m (mg)**	**$\Delta L/L_0/Q$ (C^{-1})**	**ζ (V)**
100	6.0	59.8	34.2	75.2	200.6	137.8	171.6	2.76	2.44	−0.0123	8.4
50	6.0	59.8	34.2	75.2	200.6	137.8	171.6	2.76	2.44	−0.0129	8.8
20	6.0	59.8	34.2	75.2	200.6	137.8	171.6	2.76	2.44	−0.0134	9.2
10	6.0	59.8	34.2	75.2	200.6	137.8	171.6	2.76	2.44	−0.0139	9.5
5	6.0	59.8	34.2	75.2	200.6	137.8	171.6	2.76	2.44	−0.0150	10.2
3	6.0	59.8	34.2	75.2	200.6	137.8	171.6	2.76	2.44	−0.0156	10.7

Table S2. The Young's modulus of NP-CCA was obtained based on the Young's modulus of Al, Co, and Cu metals. The Y_s value for each dealloyed sample can be estimated by such an equation: $Y(Di) = \sum X(M)*Y(M)$, (M is Al, Co and Cu).

***X*(Al) (at. %)**	***X*(Co) (at. %)**	***X*(Cu) (at. %)**	**$Y_{s(Al)}$ (GPa)**	**$Y_{s(Co)}$ (GPa)**	**$Y_{s(Cu)}$ (GPa)**	**$Y_{s(alloy)}$ (GPa)**
6.0	59.8	34.2	70.6	335.2	129.8	249.1

Table S3. Various parameters of NP-CCA and NP-AuPt. Note that the strain energy density is in linear values.

**Sample**	φ (10^{-2})	Y_s (GPa)	Y_{eff} (GPa)	ε_{max} (10^{-4})	w_M (KJ m^{-3})	w_V (KJ m^{-3})
NP-CCA	32.9	249.1	14.00	119	359.17	991.3
NP-AuPt	25.0	25.0	1.56	130	400.00	2000.0

4. References

1. Bai, Q.; Zhang, C.; Tan, F.; Zhang, Z. High-performance, low-cost nanoporous alloy actuators by one-step dealloying of Al-Ni-Cu precursors. *Intermetallics* **2022,** *145*, 107537.
2. Ouyang, B.; Peng, D.; Jiao, J.; Ye, J.; Jin, N. First-principle investigation of the stability and mechanical properties of the binder phase B(B=Co and Ni) with the maximum solubility of the transition-group elements M(M=V, Ti, Ta, Mo, W and Cr). *Mater. Today Commun.* **2023,** *34*, 105217.
